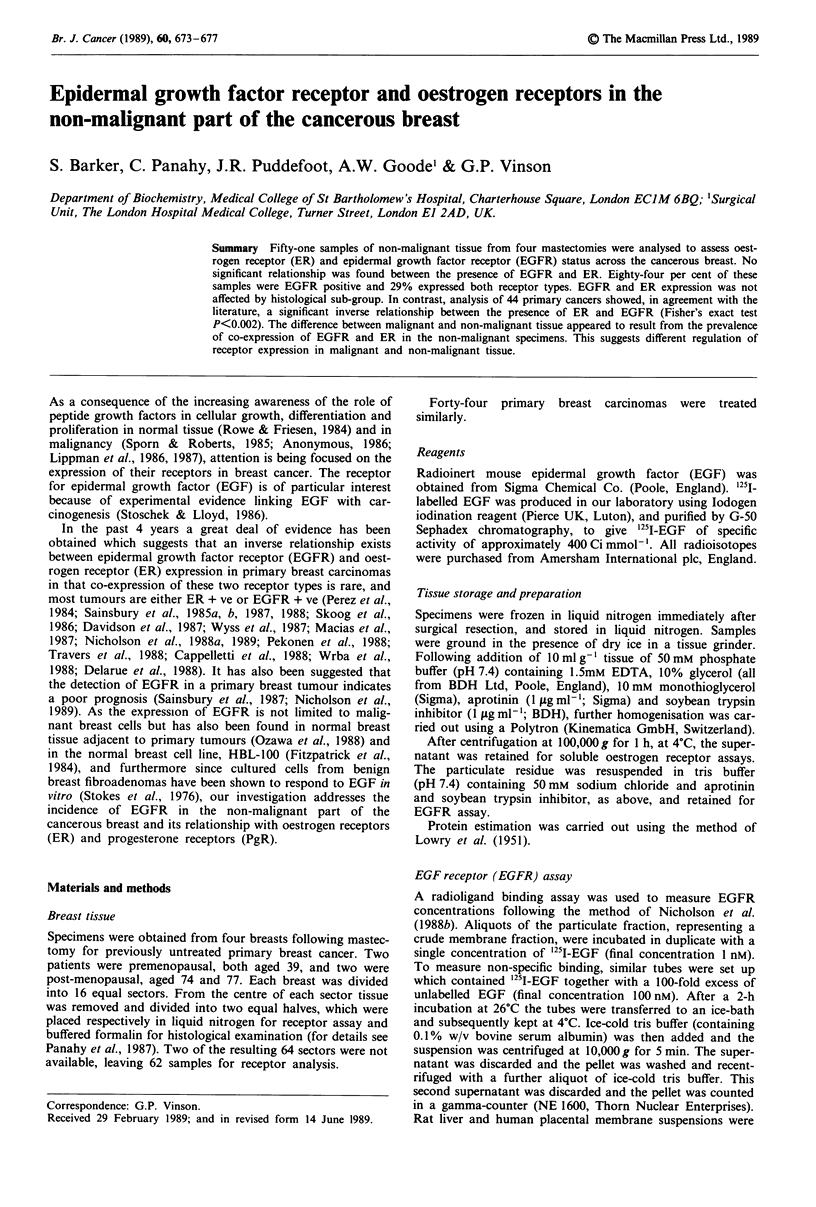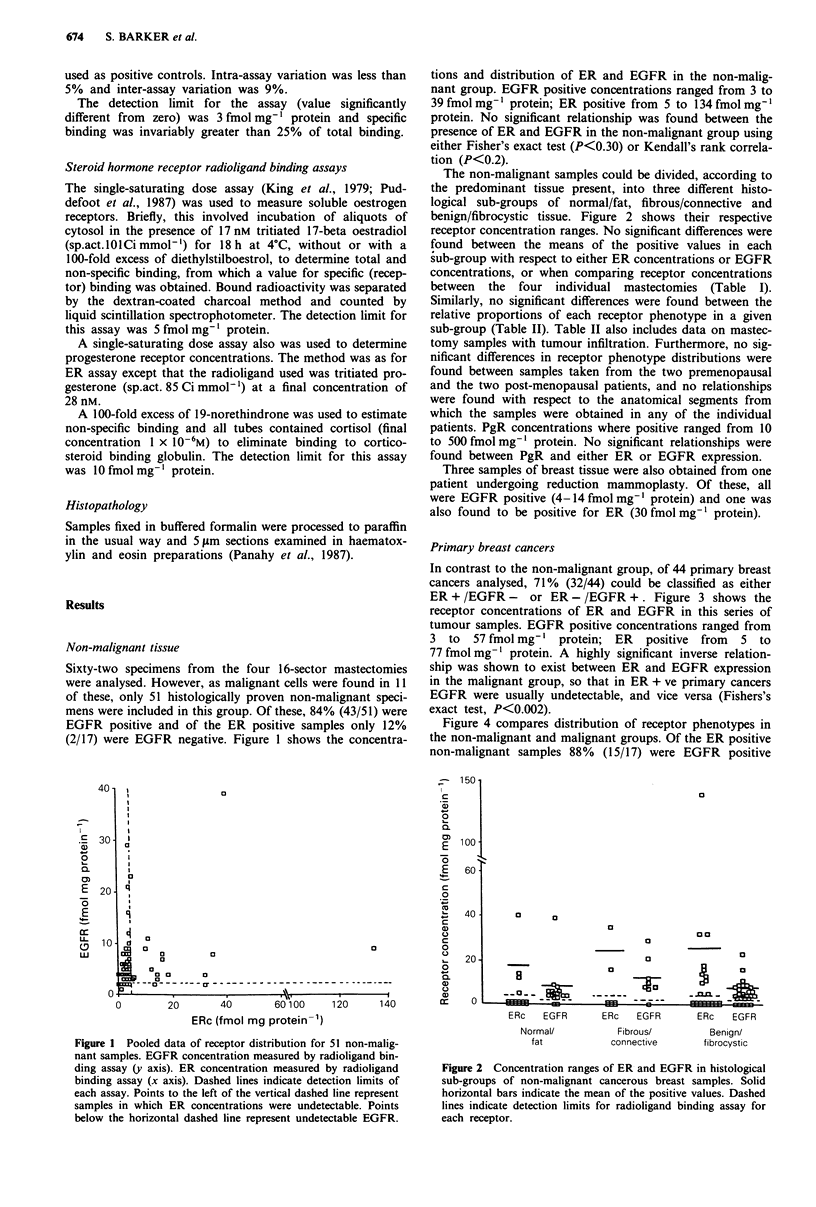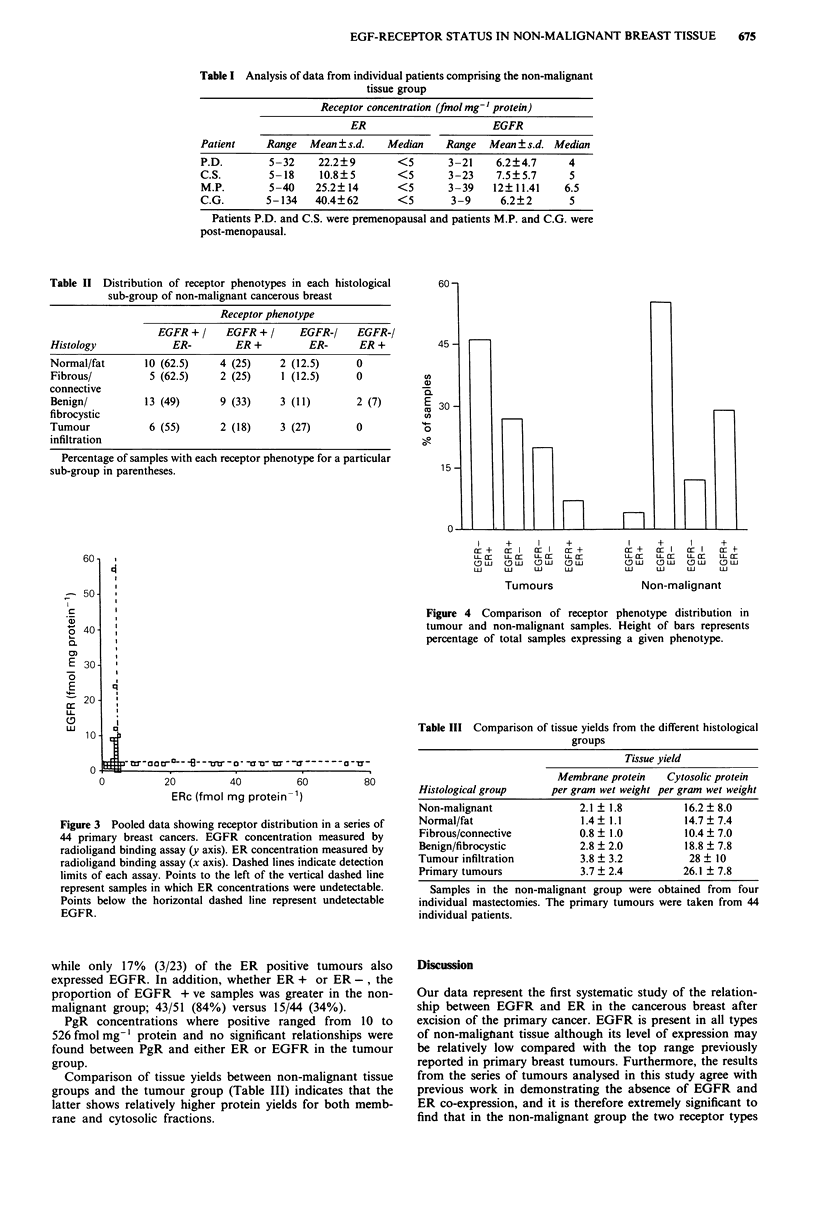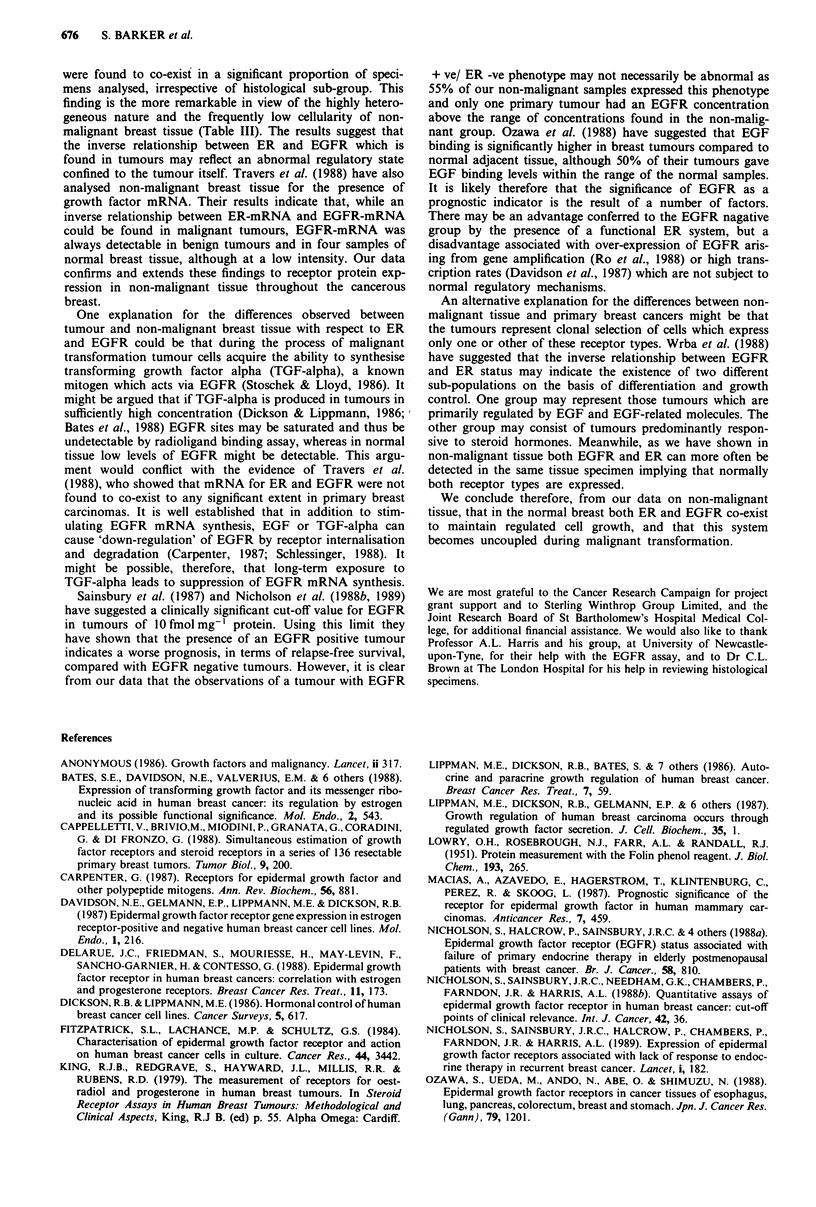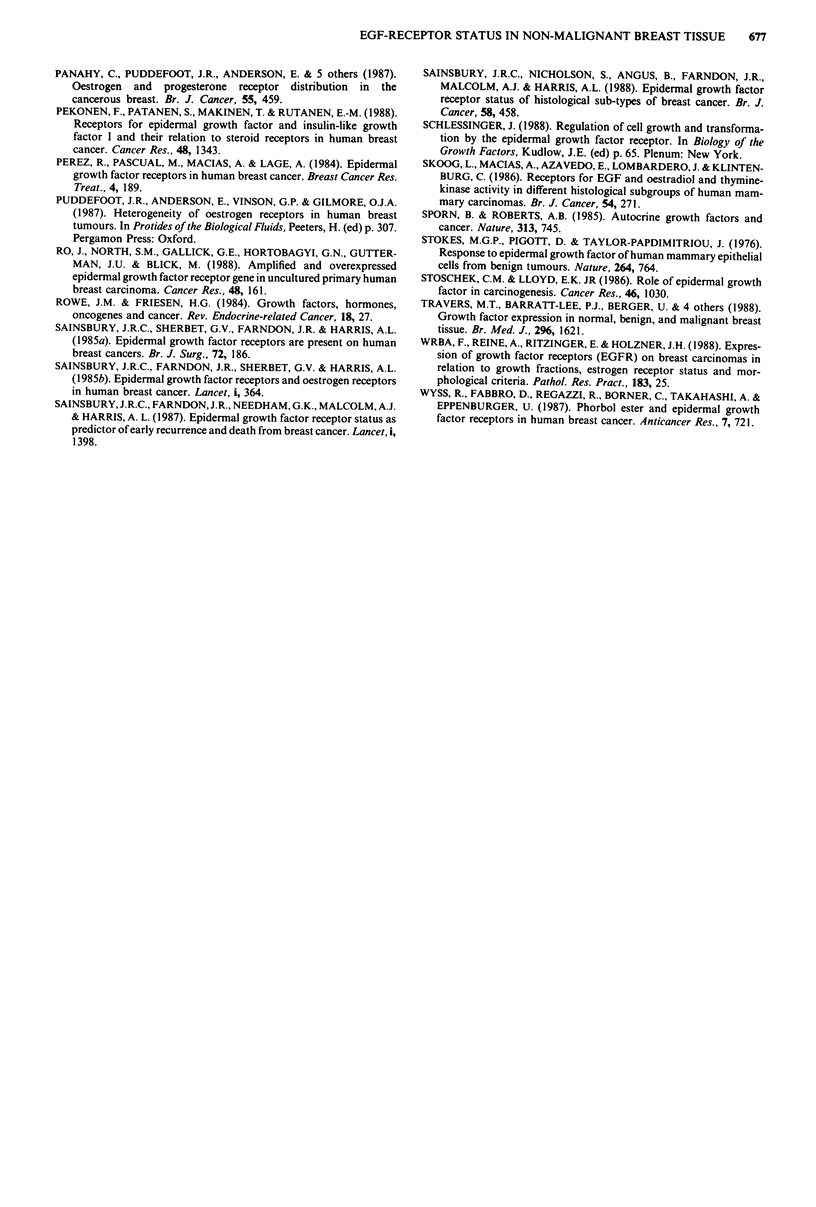# Epidermal growth factor receptor and oestrogen receptors in the non-malignant part of the cancerous breast.

**DOI:** 10.1038/bjc.1989.338

**Published:** 1989-11

**Authors:** S. Barker, C. Panahy, J. R. Puddefoot, A. W. Goode, G. P. Vinson

**Affiliations:** Department of Biochemistry, Medical College of St Bartholomew's Hospital, London, UK.

## Abstract

Fifty-one samples of non-malignant tissue from four mastectomies were analysed to assess oestrogen receptor (ER) and epidermal growth factor receptor (EGFR) status across the cancerous breast. No significant relationship was found between the presence of EGFR and ER. Eighty-four per cent of these samples were EGFR positive and 29% expressed both receptor types. EGFR and ER expression was not affected by histological sub-group. In contrast, analysis of 44 primary cancers showed, in agreement with the literature, a significant inverse relationship between the presence of ER and EGFR (Fisher's exact test P less than 0.002). The difference between malignant and non-malignant tissue appeared to result from the prevalence of co-expression of EGFR and ER in the non-malignant specimens. This suggests different regulation of receptor expression in malignant and non-malignant tissue.


Br. J. Cancer (1989), 60, 673–677 © The Macmillan Press Ltd., 1989

### Epidermal growth factor receptor and oestrogen receptors in the non-malignant part of the cancerous breast

S. Barker, C. Panahy, J.R. Puddefoot, A.W. Goode[1] & G.P. Vinson

*Department of Biochemistry, Medical College of St Bartholomew's Hospital, Charterhouse Square, London EC1M 6BQ; [1]Surgical Unit, The London Hospital Medical College, Turner Street, London E1 2AD, UK.*

**Summary** Fifty-one samples of non-malignant tissue from four mastectomies were analysed to assess oestrogen receptor (ER) and epidermal growth factor receptor (EGFR) status across the cancerous breast. No significant relationship was found between the presence of EGFR and ER. Eighty-four per cent of these samples were EGFR positive and 29% expressed both receptor types. EGFR and ER expression was not affected by histological sub-group. In contrast, analysis of 44 primary cancers showed, in agreement with the literature, a significant inverse relationship between the presence of ER and EGFR (Fisher's exact test $P<0.002$). The difference between malignant and non-malignant tissue appeared to result from the prevalence of co-expression of EGFR and ER in the non-malignant specimens. This suggests different regulation of receptor expression in malignant and non-malignant tissue.

As a consequence of the increasing awareness of the role of peptide growth factors in cellular growth, differentiation and proliferation in normal tissue (Rowe & Friesen, 1984) and in malignancy (Sporn & Roberts, 1985; Anonymous, 1986; Lippman *et al.*, 1986, 1987), attention is being focused on the expression of their receptors in breast cancer. The receptor for epidermal growth factor (EGF) is of particular interest because of experimental evidence linking EGF with carcinogenesis (Stoschek & Lloyd, 1986).

In the past 4 years a great deal of evidence has been obtained which suggests that an inverse relationship exists between epidermal growth factor receptor (EGFR) and oestrogen receptor (ER) expression in primary breast carcinomas in that co-expression of these two receptor types is rare, and most tumours are either ER + ve or EGFR + ve (Perez *et al.*, 1984; Sainsbury *et al.*, 1985*a*, *b*, 1987, 1988; Skoog *et al.*, 1986; Davidson *et al.*, 1987; Wyss *et al.*, 1987; Macias *et al.*, 1987; Nicholson *et al.*, 1988*a*, 1989; Pekonen *et al.*, 1988; Travers *et al.*, 1988; Cappelletti *et al.*, 1988; Wrba *et al.*, 1988; Delarue *et al.*, 1988). It has also been suggested that the detection of EGFR in a primary breast tumour indicates a poor prognosis (Sainsbury *et al.*, 1987; Nicholson *et al.*, 1989). As the expression of EGFR is not limited to malignant breast cells but has also been found in normal breast tissue adjacent to primary tumours (Ozawa *et al.*, 1988) and in the normal breast cell line, HBL-100 (Fitzpatrick *et al.*, 1984), and furthermore since cultured cells from benign breast fibroadenomas have been shown to respond to EGF *in vitro* (Stokes *et al.*, 1976), our investigation addresses the incidence of EGFR in the non-malignant part of the cancerous breast and its relationship with oestrogen receptors (ER) and progesterone receptors (PgR).

### Materials and methods

### *Breast tissue*

Specimens were obtained from four breasts following mastectomy for previously untreated primary breast cancer. Two patients were premenopausal, both aged 39, and two were post-menopausal, aged 74 and 77. Each breast was divided into 16 equal sectors. From the centre of each sector tissue was removed and divided into two equal halves, which were placed respectively in liquid nitrogen for receptor assay and buffered formalin for histological examination (for details see Panahy *et al.*, 1987). Two of the resulting 64 sectors were not available, leaving 62 samples for receptor analysis.

Forty-four primary breast carcinomas were treated similarly.

### *Reagents*

Radioinert mouse epidermal growth factor (EGF) was obtained from Sigma Chemical Co. (Poole, England). $^{125}$I-labelled EGF was produced in our laboratory using Iodogen iodination reagent (Pierce UK, Luton), and purified by G-50 Sephadex chromatography, to give $^{125}$I-EGF of specific activity of approximately 400 Ci mmol$^{-1}$. All radioisotopes were purchased from Amersham International plc, England.

### *Tissue storage and preparation*

Specimens were frozen in liquid nitrogen immediately after surgical resection, and stored in liquid nitrogen. Samples were ground in the presence of dry ice in a tissue grinder. Following addition of 10 ml g$^{-1}$ tissue of 50 mM phosphate buffer (pH 7.4) containing 1.5 mM EDTA, 10% glycerol (all from BDH Ltd, Poole, England), 10 mM monothioglycerol (Sigma), aprotinin (1 $\mu$g ml$^{-1}$; Sigma) and soybean trypsin inhibitor (1 $\mu$g ml$^{-1}$; BDH), further homogenisation was carried out using a Polytron (Kinematica GmbH, Switzerland).

After centrifugation at 100,000 *g* for 1 h, at 4°C, the supernatant was retained for soluble oestrogen receptor assays. The particulate residue was resuspended in tris buffer (pH 7.4) containing 50 mM sodium chloride and aprotinin and soybean trypsin inhibitor, as above, and retained for EGFR assay.

Protein estimation was carried out using the method of Lowry *et al.* (1951).

### *EGF receptor (EGFR) assay*

A radioligand binding assay was used to measure EGFR concentrations following the method of Nicholson *et al.* (1988*b*). Aliquots of the particulate fraction, representing a crude membrane fraction, were incubated in duplicate with a single concentration of $^{125}$I-EGF (final concentration 1 nM). To measure non-specific binding, similar tubes were set up which contained $^{125}$I-EGF together with a 100-fold excess of unlabelled EGF (final concentration 100 nM). After a 2-h incubation at 26°C the tubes were transferred to an ice-bath and subsequently kept at 4°C. Ice-cold tris buffer (containing 0.1% w/v bovine serum albumin) was then added and the suspension was centrifuged at 10,000 *g* for 5 min. The supernatant was discarded and the pellet was washed and recentrifuged with a further aliquot of ice-cold tris buffer. This second supernatant was discarded and the pellet was counted in a gamma-counter (NE 1600, Thorn Nuclear Enterprises). Rat liver and human placental membrane suspensions were

Correspondence: G.P. Vinson.
Received 29 February 1989; and in revised form 14 June 1989.

used as positive controls. Intra-assay variation was less than 5% and inter-assay variation was 9%.

The detection limit for the assay (value significantly different from zero) was 3 fmol $mg^{-1}$ protein and specific binding was invariably greater than 25% of total binding.

*Steroid hormone receptor radioligand binding assays*

The single-saturating dose assay (King *et al.*, 1979; Pudlefoot *et al.*, 1987) was used to measure soluble oestrogen receptors. Briefly, this involved incubation of aliquots of cytosol in the presence of 17 nM tritiated 17-beta oestradiol (sp.act.101Ci $mmol^{-1}$) for 18 h at 4°C, without or with a 100-fold excess of diethylstilboestrol, to determine total and non-specific binding, from which a value for specific (receptor) binding was obtained. Bound radioactivity was separated by the dextran-coated charcoal method and counted by liquid scintillation spectrophotometer. The detection limit for this assay was 5 fmol $mg^{-1}$ protein.

A single-saturating dose assay also was used to determine progesterone receptor concentrations. The method was as for ER assay except that the radioligand used was tritiated progesterone (sp.act. 85 Ci $mmol^{-1}$) at a final concentration of 28 nM.

A 100-fold excess of 19-norethindrone was used to estimate non-specific binding and all tubes contained cortisol (final concentration $1 \times 10^{-6}$M) to eliminate binding to corticosteroid binding globulin. The detection limit for this assay was 10 fmol $mg^{-1}$ protein.

*Histopathology*

Samples fixed in buffered formalin were processed to paraffin in the usual way and 5 µm sections examined in haematoxylin and eosin preparations (Panahy *et al.*, 1987).

### Results

*Non-malignant tissue*

Sixty-two specimens from the four 16-sector mastectomies were analysed. However, as malignant cells were found in 11 of these, only 51 histologically proven non-malignant specimens were included in this group. Of these, 84% (43/51) were EGFR positive and of the ER positive samples only 12% (2/17) were EGFR negative. Figure 1 shows the concentrations and distribution of ER and EGFR in the non-malignant group. EGFR positive concentrations ranged from 3 to 39 fmol $mg^{-1}$ protein; ER positive from 5 to 134 fmol $mg^{-1}$ protein. No significant relationship was found between the presence of ER and EGFR in the non-malignant group using either Fisher's exact test ($P<0.30$) or Kendall's rank correlation ($P<0.2$).

The non-malignant samples could be divided, according to the predominant tissue present, into three different histological sub-groups of normal/fat, fibrous/connective and benign/fibrocystic tissue. Figure 2 shows their respective receptor concentration ranges. No significant differences were found between the means of the positive values in each sub-group with respect to either ER concentrations or EGFR concentrations, or when comparing receptor concentrations between the four individual mastectomies (Table I). Similarly, no significant differences were found between the relative proportions of each receptor phenotype in a given sub-group (Table II). Table II also includes data on mastectomy samples with tumour infiltration. Furthermore, no significant differences in receptor phenotype distributions were found between samples taken from the two premenopausal and the two post-menopausal patients, and no relationships were found with respect to the anatomical segments from which the samples were obtained in any of the individual patients. PgR concentrations where positive ranged from 10 to 500 fmol $mg^{-1}$ protein. No significant relationships were found between PgR and either ER or EGFR expression.

Three samples of breast tissue were also obtained from one patient undergoing reduction mammoplasty. Of these, all were EGFR positive (4–14 fmol $mg^{-1}$ protein) and one was also found to be positive for ER (30 fmol $mg^{-1}$ protein).

*Primary breast cancers*

In contrast to the non-malignant group, of 44 primary breast cancers analysed, 71% (32/44) could be classified as either ER + /EGFR − or ER − /EGFR + . Figure 3 shows the receptor concentrations of ER and EGFR in this series of tumour samples. EGFR positive concentrations ranged from 3 to 57 fmol $mg^{-1}$ protein; ER positive from 5 to 77 fmol $mg^{-1}$ protein. A highly significant inverse relationship was shown to exist between ER and EGFR expression in the malignant group, so that in ER + ve primary cancers EGFR were usually undetectable, and vice versa (Fishers's exact test, $P<0.002$).

Figure 4 compares distribution of receptor phenotypes in the non-malignant and malignant groups. Of the ER positive non-malignant samples 88% (15/17) were EGFR positive

**Figure 1** Pooled data of receptor distribution for 51 non-malignant samples. EGFR concentration measured by radioligand binding assay (*y* axis). ER concentration measured by radioligand binding assay (*x* axis). Dashed lines indicate detection limits of each assay. Points to the left of the vertical dashed line represent samples in which ER concentrations were undetectable. Points below the horizontal dashed line represent undetectable EGFR.

**Figure 2** Concentration ranges of ER and EGFR in histological sub-groups of non-malignant cancerous breast samples. Solid horizontal bars indicate the mean of the positive values. Dashed lines indicate detection limits for radioligand binding assay for each receptor.

**Table I** Analysis of data from individual patients comprising the non-malignant tissue group

| | *Receptor concentration (fmol mg$^{-1}$ protein)* | | | | | |
|---|---|---|---|---|---|---|
| | *ER* | | | *EGFR* | | |
| *Patient* | *Range* | *Mean ± s.d.* | *Median* | *Range* | *Mean ± s.d.* | *Median* |
| P.D. | 5–32 | 22.2±9 | <5 | 3–21 | 6.2±4.7 | 4 |
| C.S. | 5–18 | 10.8±5 | <5 | 3–23 | 7.5±5.7 | 5 |
| M.P. | 5–40 | 25.2±14 | <5 | 3–39 | 12±11.41 | 6.5 |
| C.G. | 5–134 | 40.4±62 | <5 | 3–9 | 6.2±2 | 5 |

Patients P.D. and C.S. were premenopausal and patients M.P. and C.G. were post-menopausal.

**Table II** Distribution of receptor phenotypes in each histological sub-group of non-malignant cancerous breast

| | *Receptor phenotype* | | | |
|---|---|---|---|---|
| *Histology* | *EGFR + / ER-* | *EGFR + / ER +* | *EGFR-/ ER-* | *EGFR-/ ER +* |
| Normal/fat | 10 (62.5) | 4 (25) | 2 (12.5) | 0 |
| Fibrous/ connective | 5 (62.5) | 2 (25) | 1 (12.5) | 0 |
| Benign/ fibrocystic | 13 (49) | 9 (33) | 3 (11) | 2 (7) |
| Tumour infiltration | 6 (55) | 2 (18) | 3 (27) | 0 |

Percentage of samples with each receptor phenotype for a particular sub-group in parentheses.

**Figure 3** Pooled data showing receptor distribution in a series of 44 primary breast cancers. EGFR concentration measured by radioligand binding assay (*y* axis). ER concentration measured by radioligand binding assay (*x* axis). Dashed lines indicate detection limits of each assay. Points to the left of the vertical dashed line represent samples in which ER concentrations were undetectable. Points below the horizontal dashed line represent undetectable EGFR.

**Figure 4** Comparison of receptor phenotype distribution in tumour and non-malignant samples. Height of bars represents percentage of total samples expressing a given phenotype.

**Table III** Comparison of tissue yields from the different histological groups

| | *Tissue yield* | |
|---|---|---|
| *Histological group* | *Membrane protein per gram wet weight* | *Cytosolic protein per gram wet weight* |
| Non-malignant | 2.1 ± 1.8 | 16.2 ± 8.0 |
| Normal/fat | 1.4 ± 1.1 | 14.7 ± 7.4 |
| Fibrous/connective | 0.8 ± 1.0 | 10.4 ± 7.0 |
| Benign/fibrocystic | 2.8 ± 2.0 | 18.8 ± 7.8 |
| Tumour infiltration | 3.8 ± 3.2 | 28 ± 10 |
| Primary tumours | 3.7 ± 2.4 | 26.1 ± 7.8 |

Samples in the non-malignant group were obtained from four individual mastectomies. The primary tumours were taken from 44 individual patients.

while only 17% (3/23) of the ER positive tumours also expressed EGFR. In addition, whether ER + or ER – , the proportion of EGFR + ve samples was greater in the non-malignant group; 43/51 (84%) versus 15/44 (34%).

PgR concentrations where positive ranged from 10 to 526 fmol mg$^{-1}$ protein and no significant relationships were found between PgR and either ER or EGFR in the tumour group.

Comparison of tissue yields between non-malignant tissue groups and the tumour group (Table III) indicates that the latter shows relatively higher protein yields for both membrane and cytosolic fractions.

### Discussion

Our data represent the first systematic study of the relationship between EGFR and ER in the cancerous breast after excision of the primary cancer. EGFR is present in all types of non-malignant tissue although its level of expression may be relatively low compared with the top range previously reported in primary breast tumours. Furthermore, the results from the series of tumours analysed in this study agree with previous work in demonstrating the absence of EGFR and ER co-expression, and it is therefore extremely significant to find that in the non-malignant group the two receptor types were found to co-exist in a significant proportion of specimens analysed, irrespective of histological sub-group. This finding is the more remarkable in view of the highly heterogeneous nature and the frequently low cellularity of non-malignant breast tissue (Table III). The results suggest that the inverse relationship between ER and EGFR which is found in tumours may reflect an abnormal regulatory state confined to the tumour itself. Travers *et al.* (1988) have also analysed non-malignant breast tissue for the presence of growth factor mRNA. Their results indicate that, while an inverse relationship between ER-mRNA and EGFR-mRNA could be found in malignant tumours, EGFR-mRNA was always detectable in benign tumours and in four samples of normal breast tissue, although at a low intensity. Our data confirms and extends these findings to receptor protein expression in non-malignant tissue throughout the cancerous breast.

One explanation for the differences observed between tumour and non-malignant breast tissue with respect to ER and EGFR could be that during the process of malignant transformation tumour cells acquire the ability to synthesise transforming growth factor alpha (TGF-alpha), a known mitogen which acts via EGFR (Stoschek & Lloyd, 1986). It might be argued that if TGF-alpha is produced in tumours in sufficiently high concentration (Dickson & Lippmann, 1986; Bates *et al.*, 1988) EGFR sites may be saturated and thus be undetectable by radioligand binding assay, whereas in normal tissue low levels of EGFR might be detectable. This argument would conflict with the evidence of Travers *et al.* (1988), who showed that mRNA for ER and EGFR were not found to co-exist to any significant extent in primary breast carcinomas. It is well established that in addition to stimulating EGFR mRNA synthesis, EGF or TGF-alpha can cause 'down-regulation' of EGFR by receptor internalisation and degradation (Carpenter, 1987; Schlessinger, 1988). It might be possible, therefore, that long-term exposure to TGF-alpha leads to suppression of EGFR mRNA synthesis.

Sainsbury *et al.* (1987) and Nicholson *et al.* (1988*b*, 1989) have suggested a clinically significant cut-off value for EGFR in tumours of 10 fmol $mg^{-1}$ protein. Using this limit they have shown that the presence of an EGFR positive tumour indicates a worse prognosis, in terms of relapse-free survival, compared with EGFR negative tumours. However, it is clear from our data that the observations of a tumour with EGFR + ve/ ER -ve phenotype may not necessarily be abnormal as 55% of our non-malignant samples expressed this phenotype and only one primary tumour had an EGFR concentration above the range of concentrations found in the non-malignant group. Ozawa *et al.* (1988) have suggested that EGF binding is significantly higher in breast tumours compared to normal adjacent tissue, although 50% of their tumours gave EGF binding levels within the range of the normal samples. It is likely therefore that the significance of EGFR as a prognostic indicator is the result of a number of factors. There may be an advantage conferred to the EGFR nagative group by the presence of a functional ER system, but a disadvantage associated with over-expression of EGFR arising from gene amplification (Ro *et al.*, 1988) or high transcription rates (Davidson *et al.*, 1987) which are not subject to normal regulatory mechanisms.

An alternative explanation for the differences between non-malignant tissue and primary breast cancers might be that the tumours represent clonal selection of cells which express only one or other of these receptor types. Wrba *et al.* (1988) have suggested that the inverse relationship between EGFR and ER status may indicate the existence of two different sub-populations on the basis of differentiation and growth control. One group may represent those tumours which are primarily regulated by EGF and EGF-related molecules. The other group may consist of tumours predominantly responsive to steroid hormones. Meanwhile, as we have shown in non-malignant tissue both EGFR and ER can more often be detected in the same tissue specimen implying that normally both receptor types are expressed.

We conclude therefore, from our data on non-malignant tissue, that in the normal breast both ER and EGFR co-exist to maintain regulated cell growth, and that this system becomes uncoupled during malignant transformation.

We are most grateful to the Cancer Research Campaign for project grant support and to Sterling Winthrop Group Limited, and the Joint Research Board of St Bartholomew's Hospital Medical College, for additional financial assistance. We would also like to thank Professor A.L. Harris and his group, at University of Newcastle-upon-Tyne, for their help with the EGFR assay, and to Dr C.L. Brown at The London Hospital for his help in reviewing histological specimens.

### References

ANONYMOUS (1986). Growth factors and malignancy. *Lancet*, **ii** 317.

BATES, S.E., DAVIDSON, N.E., VALVERIUS, E.M. & 6 others (1988). Expression of transforming growth factor and its messenger ribonucleic acid in human breast cancer: its regulation by estrogen and its possible functional significance. *Mol. Endo.*, **2**, 543.

CAPPELLETTI, V., BRIVIO,M., MIODINI, P., GRANATA, G., CORADINI, G. & DI FRONZO, G. (1988). Simultaneous estimation of growth factor receptors and steroid receptors in a series of 136 resectable primary breast tumors. *Tumor Biol.*, **9**, 200.

CARPENTER, G. (1987). Receptors for epidermal growth factor and other polypeptide mitogens. *Ann. Rev. Biochem.*, **56**, 881.

DAVIDSON, N.E., GELMANN, E.P., LIPPMANN, M.E. & DICKSON, R.B (1987) Epidermal growth factor receptor gene expression in estrogen receptor-positive and negative human breast cancer cell lines. *Mol. Endo.*, **1**, 216.

DELARUE, J.C., FRIEDMAN, S., MOURIESSE, H., MAY-LEVIN, F., SANCHO-GARNIER, H. & CONTESSO, G. (1988). Epidermal growth factor receptor in human breast cancers: correlation with estrogen and progesterone receptors. *Breast Cancer Res. Treat.*, **11**, 173.

DICKSON, R.B. & LIPPMANN, M.E. (1986). Hormonal control of human breast cancer cell lines. *Cancer Surveys*, **5**, 617.

FITZPATRICK, S.L., LACHANCE, M.P. & SCHULTZ, G.S. (1984). Characterisation of epidermal growth factor receptor and action on human breast cancer cells in culture. *Cancer Res.*, **44**, 3442.

KING, R.J.B., REDGRAVE, S., HAYWARD, J.L., MILLIS, R.R. & RUBENS, R.D. (1979). The measurement of receptors for oestradiol and progesterone in human breast tumours. In *Steroid Receptor Assays in Human Breast Tumours: Methodological and Clinical Aspects*, King, R.J B. (ed) p. 55. Alpha Omega: Cardiff.

LIPPMAN, M.E., DICKSON, R.B., BATES, S. & 7 others (1986). Autocrine and paracrine growth regulation of human breast cancer. *Breast Cancer Res. Treat.*, **7**, 59.

LIPPMAN, M.E., DICKSON, R.B., GELMANN, E.P. & 6 others (1987). Growth regulation of human breast carcinoma occurs through regulated growth factor secretion. *J. Cell. Biochem.*, **35**, 1.

LOWRY, O.H., ROSEBROUGH, N.J., FARR, A.L. & RANDALL, R.J. (1951). Protein measurement with the Folin phenol reagent. *J. Biol. Chem.*, **193**, 265.

MACIAS, A., AZAVEDO, E., HAGERSTROM, T., KLINTENBURG, C., PEREZ, R. & SKOOG, L. (1987). Prognostic significance of the receptor for epidermal growth factor in human mammary carcinomas. *Anticancer Res.*, **7**, 459.

NICHOLSON, S., HALCROW, P., SAINSBURY, J.R.C. & 4 others (1988*a*). Epidermal growth factor receptor (EGFR) status associated with failure of primary endocrine therapy in elderly postmenopausal patients with breast cancer. *Br. J. Cancer.*, **58**, 810.

NICHOLSON, S., SAINSBURY, J.R.C., NEEDHAM, G.K., CHAMBERS, P., FARNDON, J.R. & HARRIS, A.L. (1988*b*). Quantitative assays of epidermal growth factor receptor in human breast cancer: cut-off points of clinical relevance. *Int. J. Cancer*, **42**, 36.

NICHOLSON, S., SAINSBURY, J.R.C., HALCROW, P., CHAMBERS, P., FARNDON, J.R. & HARRIS, A.L. (1989). Expression of epidermal growth factor receptors associated with lack of response to endocrine therapy in recurrent breast cancer. *Lancet*, **i**, 182.

OZAWA, S., UEDA, M., ANDO, N., ABE, O. & SHIMUZU, N. (1988). Epidermal growth factor receptors in cancer tissues of esophagus, lung, pancreas, colorectum, breast and stomach. *Jpn. J. Cancer Res. (Gann)*, **79**, 1201.

PANAHY, C., PUDDEFOOT, J.R., ANDERSON, E. & 5 others (1987). Oestrogen and progesterone receptor distribution in the cancerous breast. *Br. J. Cancer*, **55**, 459.

PEKONEN, F., PATANEN, S., MAKINEN, T. & RUTANEN, E.-M. (1988). Receptors for epidermal growth factor and insulin-like growth factor I and their relation to steroid receptors in human breast cancer. *Cancer Res.*, **48**, 1343.

PEREZ, R., PASCUAL, M., MACIAS, A. & LAGE, A. (1984). Epidermal growth factor receptors in human breast cancer. *Breast Cancer Res. Treat.*, **4**, 189.

PUDDEFOOT, J.R., ANDERSON, E., VINSON, G.P. & GILMORE, O.J.A. (1987). Heterogeneity of oestrogen receptors in human breast tumours. In *Protides of the Biological Fluids*, Peeters, H. (ed) p. 307. Pergamon Press: Oxford.

RO, J., NORTH, S.M., GALLICK, G.E., HORTOBAGYI, G.N., GUTTERMAN, J.U. & BLICK, M. (1988). Amplified and overexpressed epidermal growth factor receptor gene in uncultured primary human breast carcinoma. *Cancer Res.*, **48**, 161.

ROWE, J.M. & FRIESEN, H.G. (1984). Growth factors, hormones, oncogenes and cancer. *Rev. Endocrine-related Cancer*, **18**, 27.

SAINSBURY, J.R.C., SHERBET, G.V., FARNDON, J.R. & HARRIS, A.L. (1985*a*). Epidermal growth factor receptors are present on human breast cancers. *Br. J. Surg.*, **72**, 186.

SAINSBURY, J.R.C., FARNDON, J.R., SHERBET, G.V. & HARRIS, A.L. (1985*b*). Epidermal growth factor receptors and oestrogen receptors in human breast cancer. *Lancet*, **i**, 364.

SAINSBURY, J.R.C., FARNDON, J.R., NEEDHAM, G.K., MALCOLM, A.J. & HARRIS, A. L. (1987). Epidermal growth factor receptor status as predictor of early recurrence and death from breast cancer. *Lancet*, **i**, 1398.

SAINSBURY, J.R.C., NICHOLSON, S., ANGUS, B., FARNDON, J.R., MALCOLM, A.J. & HARRIS, A.L. (1988). Epidermal growth factor receptor status of histological sub-types of breast cancer. *Br. J. Cancer*, **58**, 458.

SCHLESSINGER, J. (1988). Regulation of cell growth and transformation by the epidermal growth factor receptor. In *Biology of the Growth Factors*, Kudlow, J.E. (ed) p. 65. Plenum: New York.

SKOOG, L., MACIAS, A., AZAVEDO, E., LOMBARDERO, J. & KLINTENBURG, C. (1986). Receptors for EGF and oestradiol and thyminekinase activity in different histological subgroups of human mammary carcinomas. *Br. J. Cancer*, **54**, 271.

SPORN, B. & ROBERTS, A.B. (1985). Autocrine growth factors and cancer. *Nature*, **313**, 745.

STOKES, M.G.P., PIGOTT, D. & TAYLOR-PAPDIMITRIOU, J. (1976). Response to epidermal growth factor of human mammary epithelial cells from benign tumours. *Nature*, **264**, 764.

STOSCHEK, C.M. & LLOYD, E.K. JR (1986). Role of epidermal growth factor in carcinogenesis. *Cancer Res.*, **46**, 1030.

TRAVERS, M.T., BARRATT-LEE, P.J., BERGER, U. & 4 others (1988). Growth factor expression in normal, benign, and malignant breast tissue. *Br. Med. J.*, **296**, 1621.

WRBA, F., REINE, A., RITZINGER, E. & HOLZNER, J.H. (1988). Expression of growth factor receptors (EGFR) on breast carcinomas in relation to growth fractions, estrogen receptor status and morphological criteria. *Pathol. Res. Pract.*, **183**, 25.

WYSS, R., FABBRO, D., REGAZZI, R., BORNER, C., TAKAHASHI, A. & EPPENBURGER, U. (1987). Phorbol ester and epidermal growth factor receptors in human breast cancer. *Anticancer Res.*, **7**, 721.